# Mode of Action of Neonicotinoid Insecticides Imidacloprid and Thiacloprid to the Cockroach Pameα7 Nicotinic Acetylcholine Receptor

**DOI:** 10.3390/ijms22189880

**Published:** 2021-09-13

**Authors:** Alison Cartereau, Emiliane Taillebois, Jean-Yves Le Questel, Steeve H. Thany

**Affiliations:** 1 Université d’Orléans, LBLGC USC INRAE 1328, 45067 Orléans, France; alison.cartereau@univ-orleans.fr (A.C.); emiliane.taillebois@univ-orleans.fr (E.T.); 2 Université de Nantes, CNRS, CEISAM UMR 6230, 44000 Nantes, France

**Keywords:** acetylcholine, nicotinic receptors, neonicotinoids, imidacloprid, thiacloprid, molecular modeling, binding studies

## Abstract

The functional expression of the cockroach Pameα7 nicotinic acetylcholine receptor subunit has been previously studied, and was found to be able to form a homomeric receptor when expressed in *Xenopus laevis* oocytes. In this study, we found that the neonicotinoid insecticide imidacloprid is unable to activate the cockroach Pameα7 receptor, although thiacloprid induces low inward currents, suggesting that it is a partial agonist. In addition, the co-application or 5 min pretreatment with 10 µM imidacloprid increased nicotine current amplitudes, while the co-application or 5 min pretreatment with 10 µM thiacloprid decreased nicotine-evoked current amplitudes by 54% and 28%, respectively. This suggesting that these two representatives of neonicotinoid insecticides bind differently to the cockroach Pameα7 receptor. Interestingly, the docking models demonstrate that the orientation and interactions of the two insecticides in the cockroach Pameα7 nAChR binding pocket are very similar. Electrophysiological results have provided evidence to suggest that imidacloprid and thiacloprid could act as modulators of the cockroach Pameα7 receptors.

## 1. Introduction

Nicotinic acetylcholine receptors (nAChRs) are representative members of the Cys-loop ligand-gated ion channels (LGICs) superfamily, alongside GABA, glycine, and 5-HT3 receptors [1,2,3]. These neuroreceptors are essential for synaptic transmission processes in both vertebrate and insect nervous systems, receiving the chemical signals between neurons in the nervous system and converting them into electrical outputs. nAChRs are pentameric LGICs, with the five subunits being symmetrically or pseudosymmetrically arranged around a central ion-conducting pore, from homo- or heteropentamers of the related subunits [1,2,3]. These properties have made nAChRs important targets for human and veterinary drugs, as well as insecticides. However, the functional organization of nAChRs, as well as their diversity in terms of subunit composition and stoichiometry is much better understood in vertebrates than insects [4,5,6]. Pharmacological studies show that there at least two pharmacologically distinct classes of nAChRs α-bungarotoxin (α-Bgt)-sensitive and -insensitive, and as in vertebrates, insect α7-like subunits are potential candidates to form α-Bgt-sensitive receptors. Insect neuronal nAChRs are of particular interest because they are the main target of neonicotinoid insecticides, which are important in agriculture and veterinary medicine for controlling insect pests and preventing the transmission of insect borne diseases [7,8,9]. In general, the pharmacological properties of insect nAChRs are studied using electrophysiological approaches with isolated neurons expressing nAChR subtypes. In particular, cockroach neurons from thoracic ganglia and dorsal unpaired median (DUM) neurons have been used as models to characterize the pharmacological properties of insect native nAChR subtypes [10,11,12,13]. Using cockroach thoracic ganglia, two α-Bgt-sensitive nAChR subtypes were characterized as desensitizing (nAChD), which are selectively inhibited by imidacloprid (IMI), and non-desensitizing (nAChN), which are selectively inhibited by methyllycaconitine [10,14]. Moreover, nAChD receptors have been shown to be significantly inhibited by neonicotinoid insecticides, while in contrast, nAChN are activated by neonicotinoids [10]. α-Bgt-sensitive and -insensitive nAChR subtypes were also found in the DUM neurons. Two α-Bgt-insensitive receptors were identified as nAChR1, which are sensitive to IMI and selectively blocked by d-tubocurarine (d-TC), while nAChR2 is inhibited by mecamylamine [13,15,16,17]. Together, these studies have been used to monitor and analyze the mode of action of neonicotinoid insecticides on insect nAChRs, in order to understand the mechanisms of resistance.

In the aim of characterizing the nAChR subunits involved in the pharmacological properties of cockroach native nAChR subtypes, eight nAChR subunits (seven α and one β subunits) were cloned in the cockroach *Periplaneta americana* [18], while around ten subunits were found in other insect species [19,20,21,22,23]. Indeed, no *Periplaneta americana* α5 (Pameα5) subunit was identified in the cockroach nervous system, suggesting that the cockroach lacked this subunit. Pameα6 and Pameα7 form a cluster with drosophila Dα7 while Pameα8 is close to drosophila Dβ2, suggesting that it corresponds more with a β rather than to an α subunit [18]. The inhibition of cockroach Pameα3, Pameα8 and Pameβ1 expression by antisense oligonucleotides reduced IMI and nicotine current amplitudes, which suggests that they could be included in nAChR1 subtype. Whereas, the inhibition of Pameα1, Pameα2 and Pameβ1 decreased nicotine-activated currents, which correspond to the current-voltage curve of nAChR2 subtype [24]. Moreover, the specific inhibition of the cockroach Pameβ1 subunit resulted in a decrease in nicotine-induced currents, confirming that this subunit accounts for functional nAChR subtypes [18]. These data suggest that more than three neuronal nAChR subtypes were expressed in the cockroach DUM neurons [24]. Based on the number of subunits, and given that the pharmacological properties of these receptors were recorded using whole-cell patch-clamp recording, we hypothesize that nAChD, nAChN, nAChR1 and nAChR2 may be considered as receptor populations with different neuronal nAChR subtypes. While the nature of the subunits has been shown to be crucial in the assembly and function of heteromeric receptors [25], this study aimed to examine the relevant electrophysiological and structural properties of a homomeric cockroach Pameα7 nAChR.

**Figure 1:**
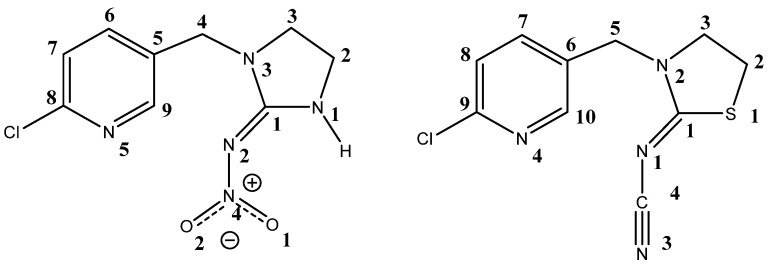
Chemical structures of the neonicotinoids, imidacloprid (**left**) and thiacloprid (**right**).

We examined the voltage-clamp recordings and molecular docking studies of the interactions of two relevant representatives of neonicotinoid insecticides (Figure 1), IMI and thiacloprid (THI), with cockroach homomeric Pameα7 nAChR [26]. We demonstrated that the orientation and interactions of the two insecticides in the Pameα7 nAChR binding pocket are very similar. The relative contributions of the various amino acid residues composing the binding site are presented and discussed, according to the nature of the neonicotinoid.

## 2. Results and Discussion

### 2.1. Effect of Neonicotinoid Insecticides on Nicotine-Induced Currents

**Figure 2:**
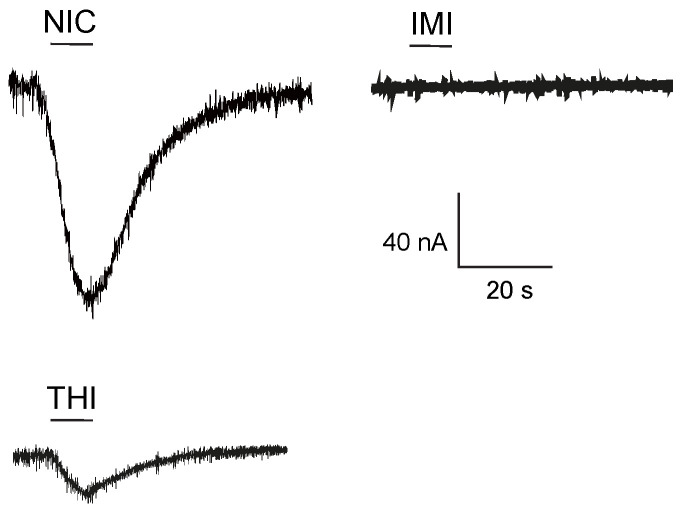
Currents induced by nicotine (NIC), imidacloprid (IMI) and thiacloprid (THI) on Pame*α*7 homomeric receptors expressed on *Xenopus laevis* oocytes.

We previously demonstrated that cockroach nervous system expressed homomeric Pame*α*7 nAChR [26]. To gain a better understanding of the functional properties of cockroach Pame*α*7 nAChRs, we have studied the effect of neonicotinoid insecticides, since they have been understood to be agonists of cockroach nAChRs [12,27]. Here, we found that IMI did not induce inward currents on Pame*α*7 nAChRs. No macroscopic currents were detected within the recorded limits of detection (Figure 2). The lack of agonist action with IMI is not surprising as it is considered a partial agonist of cockroach [12,15] and honeybee nAChRs [28]. Therefore, it may not activate the cockroach Pame*α*7 receptor, but as suggested, could activate a cockroach receptor composed of Pame*α*3, Pame*α*8 and Pame*β*1 subunits [24]. Indeed, the blocking expression of these subunits caused a decrease in both nicotine and IMI-induced current amplitudes [24]. Interestingly, THI was able to induce inward currents which were 17% of the nicotine-evoked current amplitudes (Figure 2).

**Figure 3:**
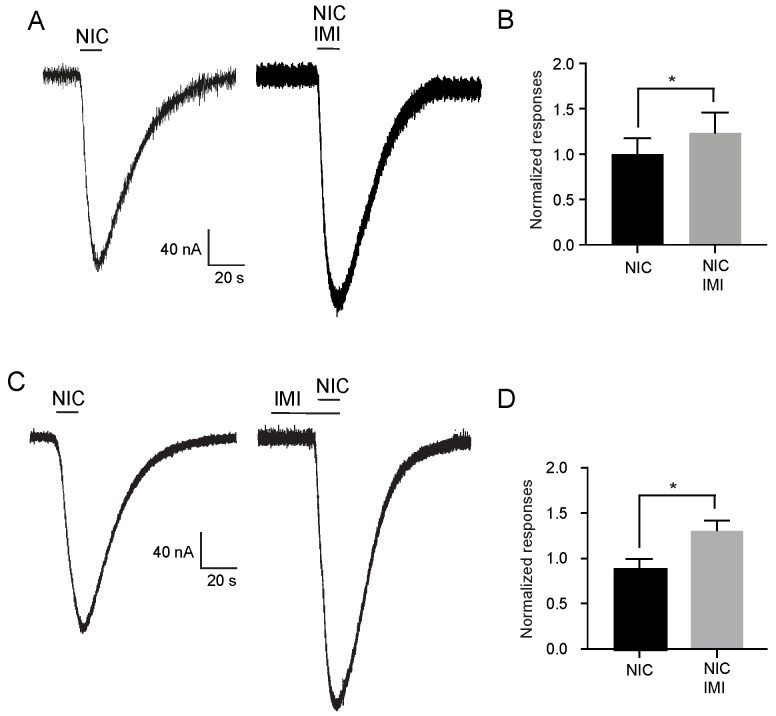
Effect of co-application and pretreatment of IMI on nicotine-evoked current amplitudes. (**A**) Currents induced by 10 mM nicotine (NIC) and the co-application of both 10 µM imidacloprid and 10 mM nicotine. (**B**) Histograms illustrating co-application of 10 µM imidacloprid (IMI) with 10 mM nicotine. (**C**,**D**) currents and histograms under 10 mM nicotine and 5 min pretreatment with 10 µM imidacloprid (IMI). Data are mean ± SEM. * *p* < 0.05. Each histogram represents *n* = 12.

We proposed that IMI and THI showed distinct effects on the cockroach Pame*α*7 receptor. THI was a partial agonist of the Pame*α*7 receptor, while IMI showed no agonist effect. Moreover, analysis of the modulatory effect induced by both IMI and THI revealed that co-application or 5 min pretreatment of 10 µM IMI with 10 mM nicotine had a significant effect on nicotine current amplitudes, IMI increased nicotine-evoked current amplitudes to 30% and 40%, respectively (*p* < 0.05, *n* = 12, Figure 3). Moreover, no statistical difference was found between 5 min pretreatment and co-application of IMI (One-way ANOVA, *p* > 0.05, *n* = 10).

**Figure 4:**
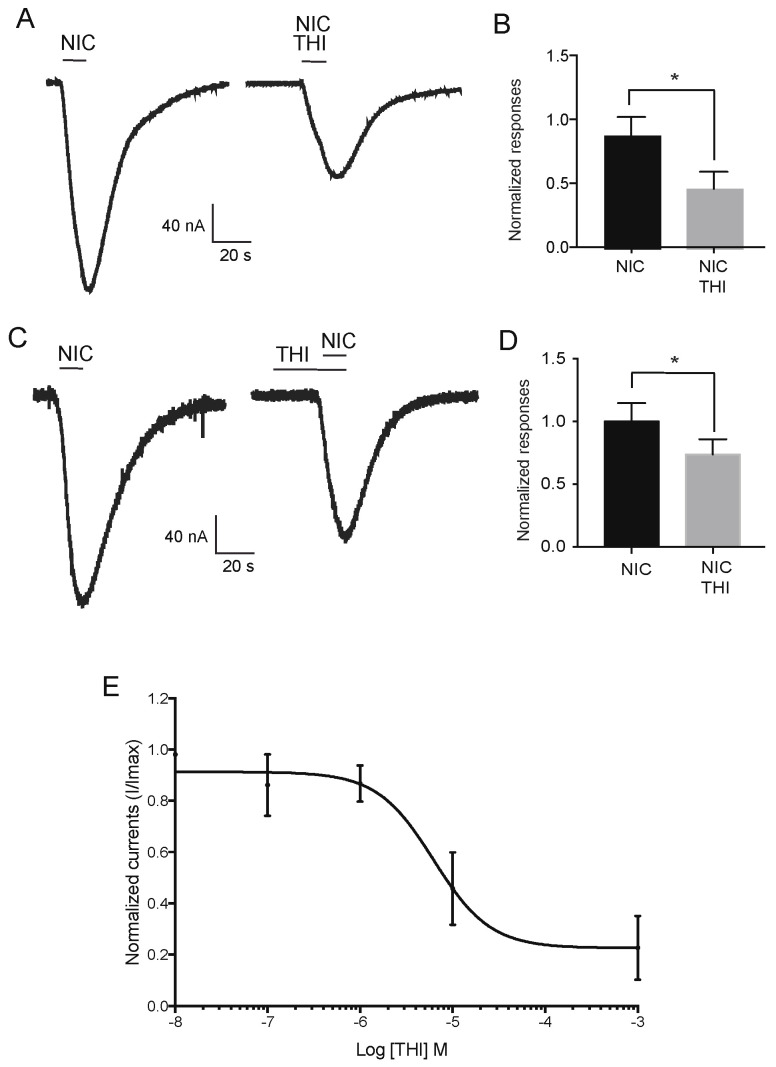
Effects of co-application or pretreatment of THI on nicotine-evoked currents. (**A**,**B**) show the effect of co-application of 10 µM THI on currents induced by bath application of 10 mM nicotine. (**C**,**D**) illustrate currents-evoked by 10 mM nicotine after pretreatment with 10 µM THI. In all histograms, data are mean ± SEM, *n* = 6 and * *p* < 0.05. Note that no significant difference was found when in the THI effects with or without 5 min pretreatment. While, (**E**) represents the inhibition-response curve under co-application of THI with 10 mM nicotine. Each point represents *n* = 6.

The co-application of 10 µM THI with 10 mM nicotine significantly decreased nicotine-induced current amplitudes to 54% of the control currents when they were co-applied (*p* < 0.05, *n* = 6, Figure 4A,B), or 28% after 5 min pretreatment (*p* < 0.05, *n* = 6, Figure 4C,D) with 10 µM THI. These results confirm that the two neonicotinoids could be divided into two subgroups having distinct effects on the Pame*α*7 receptor.

### 2.2. Sequence Alignments and Homology Modeling

**Figure 5:**
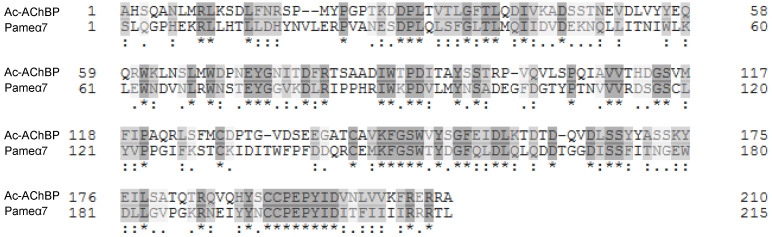
Sequence alignment of Ac-AChBP and cockroach Pameα7 receptor used to generate the homology model. (*) indicate conserved residues. (:) and (.) indicate conserved and semi-conserved substitutions.

**Figure 6:**
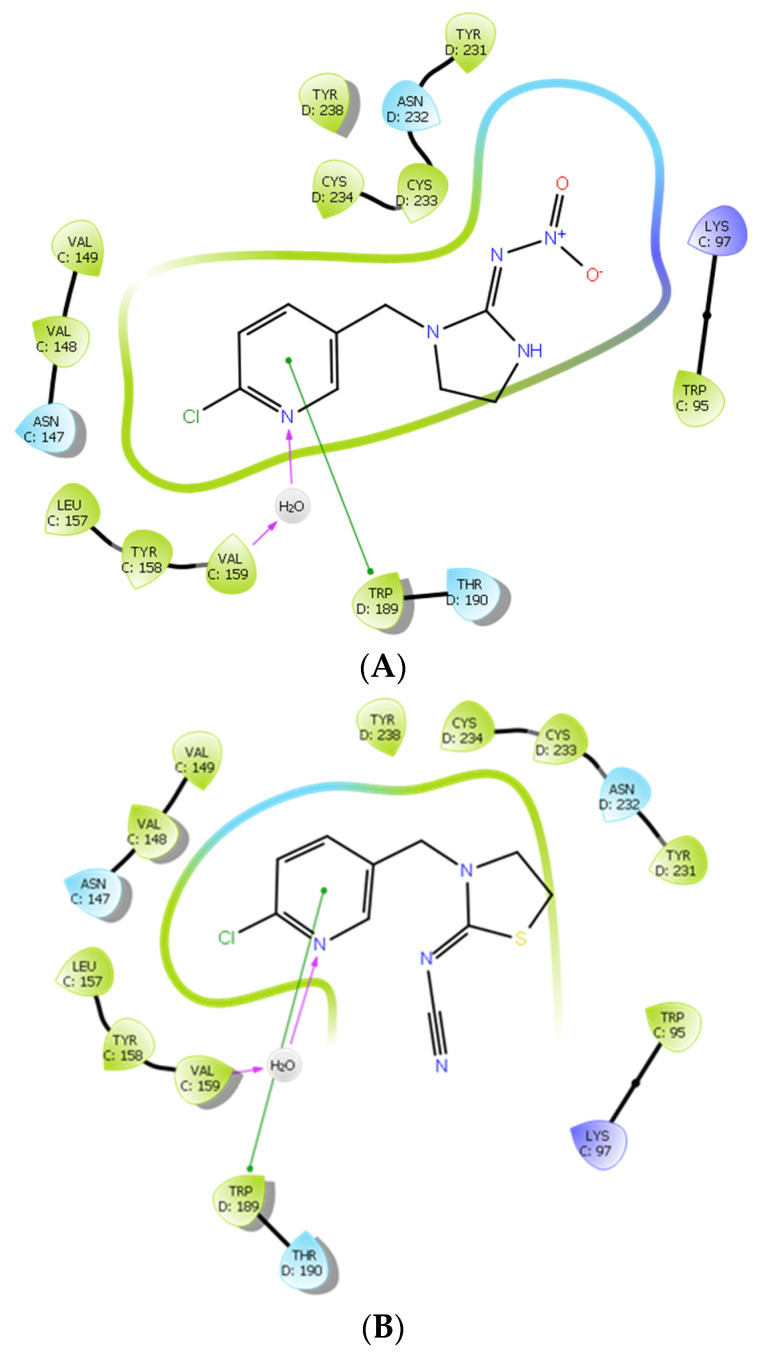
2D interaction diagrams of the ligand-receptor interactions established within the CD interface of the cockroach Pameα7 receptor for (**A**) IMI and (**B**) THI.

**Figure 7:**
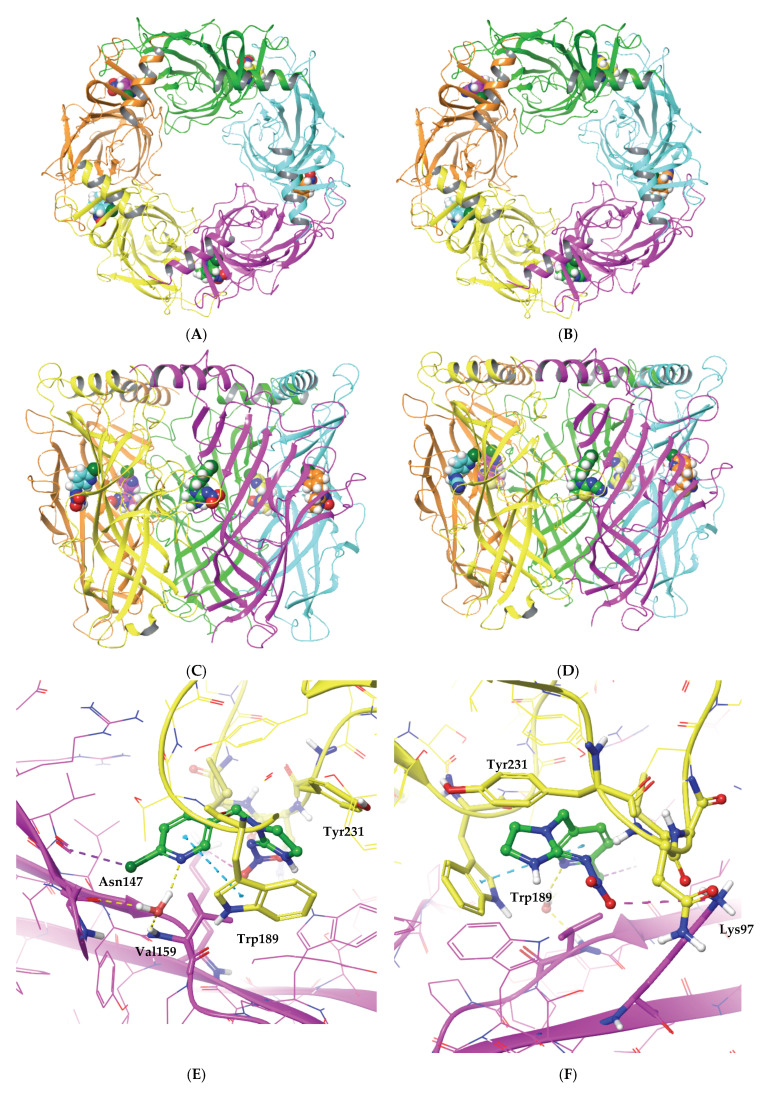

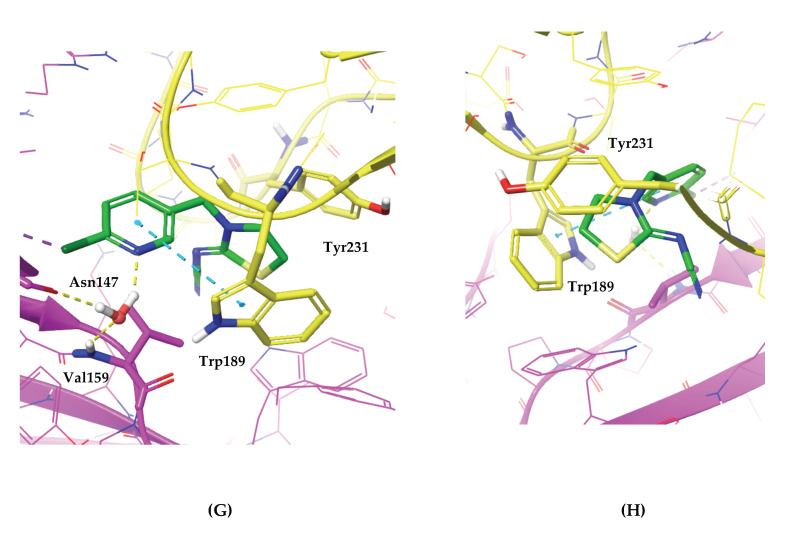
Predicted binding modes of IMI and THI with the cockroach Pameα7 receptor viewed according to four orientations: from the top of the homomeric pentameric nAChR (**top** of the **A**,**B**); from the side of the C and D subunits (**C**,**D**); with the pyridinic ring (**left**: **E**,**G**) and the push-pull fragment (**right**: **F**,**H**), in the plane of the figure, respectively. The docking scores and glide energies computed were −7.2 and −75.1 kcal/mol for IMI and −4.6 and −71.7 kcal/mol for THI, respectively. For clarity, the labelling of the key amino acids involved in intermolecular interactions with the two ligands have been added to (**E**–**G**), with these amino acids being represented as ball-and-stick models. For the same reasons, only the polar hydrogen atoms are represented. Tyr: tyrosine, Trp: tryptophan, Val: valine, Asn: asparagine, Lys: lysine.

The pairwise sequence alignments of the extracellular domain of the cockroach Pameα7 nAChR subunits against the template crystal structure (PDB ID: 3C79) [29] show 28.4% identity and 36.7% similarity, respectively (Figure 5). The sequence identity value appears slightly below the 30% “threshold” for high accuracy template-based 3D modeling. However, despite this relatively low level of sequence identity, accurate alignment can be obtained in this case as reflected by the sequence similarity value. Based on the sequence alignments, the 3D models of cockroach Pameα7 nAChR homo-pentamers were built using the Prime module [30] of the Schrodinger suite 2014-1 [31] (Figure 6 and Figure 7).

The generated models were energy minimized using the OPLS-2005 force field [32]. The quality of the 3D model, in terms of geometry was assessed using the MolProbity program [33]. Both the final models showed 99.6% residues (except proline and glycine residues), with their φ,ψ torsional angles in the most energetically favored regions of the Ramachandran plot [34]. The protein backbone root mean square deviations (RMSD) of the final cockroach Pameα7 nAChR model was of 0.38Å, using the template structure (3C79) as the reference, and no deviation was observed in the important loops within the binding site.

### 2.3. Investigating the Binding of Imidacloprid and Thiacloprid to the Cockroach Pameα7nAChR through Molecular Docking Studies

In order to determine the binding interactions of neonicotinoids to the homology models of the cockroach Pameα7 nAChR at the atomic level, we carried out docking studies with relevant neonicotinoids. We selected IMI and THI for these investigations, given that; (i) they are forerunners of two chemical classes of neonicotinoid, namely nitro- and cyano-substituted neonicotinoids; (ii) their behavior was notably different from an electrophysiological point of view. The same trends were observed in all five interfaces, and only the results obtained for one of the subunits (CD) will be presented and discussed here.

The geometric and docking parameters are reported in Table S1 of the Supplementary Materials Section. Figure 6A,B show the 2D interaction diagrams of IMI and THI respectively bound to the cockroach Pameα7 nAChR. Whereas, Figure 7 shows complementary 3D views of the predicted binding modes of the two neonicotinoids.

The docking scores and glide energies of the top ranked poses show very similar trends for both IMI and THI insecticides (Table S1, Supplementary Materials and Figure 7). Indeed, the slight preference for IMI evident for the two parameters (of about 2.5 and 3 kcal mol^−^^1^ for docking score and glide energy, respectively) must not be used to draw conclusions and should be considered cautiously. Nevertheless, such studies allow the key interactions established between the IMI and THI insecticides and their target to be delineated. Figure 7A–D show top and side views of the homomeric cockroach Pameα7 nAChR, allowing the location of the ligand binding site to be visualized at the interface between two subunits. Figure 7E–H show the key interactions established between each neonicotinoid and the binding site amino acids. Therefore, the pyridine ring of the two ligands forms π-π interactions with Trp189 of the cockroach Pameα7 nAChR. In addition, and in agreement with the trends observed in the crystallographic structures of neonicotinoid-AChBP complexes [29,35,36,37,38,39,40,41,42], the pyridine ring is consistently involved in water-mediated HB interactions with the main chain groups of amino acid residues, and in the case of the cockroach Pameα7 nAChR, corresponding to Asn147 (CO) and Val159 (NH) (Figure 7). Comparable trends were obtained for the end groups of the push-pull fragment of the two ligands, given that no specific contact is predicted between this moiety of the two insecticides and the receptor residues. In fact, in concordance with experimental data, the stability of the two ligands inside the binding pocket appears to be realized through CH…π and π…π interactions, which involve positively polarized CH groups of the five membered saturated ring of the ligands and aromatic amino acid residues of the cockroach Pameα7 nAChR, such as Trp189 and Tyr231 (see Table S1 in Supplementary Materials). It is also worth noting that short contacts are established between the ligand and one sulfur atom of Cys233. These trends are consistent with the key role played by Trp and dicysteine residues in the binding of neonicotinoids insecticides to insect nAChR models.

## 3. Conclusions

Using electrophysiological measurements based on voltage-clamp recordings, we found that, as opposed to THI, IMI could not activate cockroach Pameα7 nAChRs, as no macroscopic currents were detected in our recording conditions. Our results are compatible with the neonicotinoids’ modulatory effect on nicotine-induced currents. Indeed, we found that THI decreases nicotine evoked current amplitudes, while IMI induces an increase. Nevertheless, we cannot exclude that IMI has a weak partial agonist action, which was not detected in our experimental conditions. In the same way, the decrease in nicotine-evoked currents under bath application of THI could be associated to a competitive effect on the binding site. Indeed, we found a strong modulatory effect under co-application, which was not present with pre-treatment. Complementary molecular docking investigations led to consistent results with the currently available structural data for the interactions of IMI and THI with 3D nAChR models. Therefore, it provides a starting point for analyzing the relative behavior of neonicotinoid insecticides to cockroach Pameα7 homomeric receptor at the atomic level. The trends that have been highlighted by the electrophysiological results, which suggest that IMI and THI are considered to be positive and negative modulators of the cockroach Pameα7 receptor, respectively. Further electrophysiological, molecular modeling and binding studies on these compounds and other representatives of neonicotinoid insecticides (acetamiprid, clothianidin, thiametoxam) are ongoing in our groups. An exciting prospect for Pameα7 nAChR is to confirm the importance of amino acid residues in the ACh binding domain, particularly in the loop B-C interval in the neonicotinoid selectivity. Finally, the present data reinforce our findings that neonicotinoid insecticides, including nitro- and cyano-substituted neonicotinoids act as agonists and/or modulators of insect homomeric nAChR subtypes. Moreover, insect α-7-like receptors could be used to analyze and compare the binding properties of neonicotinoid insecticides, similar to studies conducted with hybrid receptors.

## 4. Materials and Methods

### 4.1. Compounds

ACh, nicotine, IMI, THI and α-Bgt were purchased from Sigma Chemical Co, (St Quentin, France).

### 4.2. Oocytes Injection

*Xenopus laevis* oocytes were purchased from the University of Rennes, France (CRB Xenope, Rennes, France) and kept in standard oocyte saline (SOS) of the following composition: in mM, 100 NaCl, 2 KCl, 1 MgCl_2_, 1.8 CaCl_2_ and 5 HEPES, pH 7.5. Stages V and VI oocytes were harvested and defolliculated after treatment with 2 mg·mL^−1^ collagenase IA (Sigma, Lezennes, France) in Ca^2+^-free SOS solution, supplemented with 0.8 mg·mL^−1^ trypsin inhibitor. Defolliculated oocytes were injected into nuclei with 2 ng of α7 cDNA cloned in pSGEM [43,44,45]. Injected oocytes were maintained at 18 °C in SOS solution supplemented with penicillin (100 U·mL^−1^), streptomycin (100 mg·mL^−1^), gentamycin (50 mg·mL^−1^) and sodium pyruvate (2.5 mM).

### 4.3. Voltage-Clamp Recordings

Currents were recorded 4 days after injection, using two microelectrodes filled with 3 M KCl. The oocyte membrane potential was held at −80 mV [46], and perfused continuously with recording buffer at room temperature (20–22 °C). To suppress any possible endogenous muscarinic responses, saline containing 0.5 μM atropine (Sigma, Saint Quentin, France) was employed [47,48]. Oocytes were challenged with a test compound for 5 min intervals to minimize receptor desensitization [44]. To evaluate neonicotinoids modulation of nicotine responses, they were tested with and without 5 min pretreatment. Experimental data were digitized with a Digidata−1322A A/D converter and later analyzed with pCLAMP (Molecular Devices, Union City, CA, USA). All compound solutions were prepared with the SOS.

### 4.4. Homology Modeling

The amino acid sequence of the cockroach *Periplaneta americana* Pameα7 subunit were extracted from the Uniprot server (www.expasy.org, access date: 15 July 2021) [49]. The nearest homology of the target sequences were characterized using the BLAST program [50]. The crystal structure of the acetylcholine binding protein (Ac-AChBP) extracted from *Aplysia californica* (Ac) (PDB ID: 3C79) [35] was selected as a template to build the three-dimensional (3D) model of cockroach nAChRs. Indeed, Ac-AChBP is recognized as the surrogate of the ligand binding domain of the extracellular domain of insect nAChRs [35]. The crystal structures of the Ac-AChBP-neonicotinoid complexes (PDB ID: 3C79 and PDB ID: 3C84 for IMI and THI ligands, respectively) were downloaded from the Protein Data Bank (www.pdb.org) [35]. The pairwise sequence alignments were performed to align the target and the template sequence. The 3D homology models were built using the Prime v3.6 (Schrödinger, LLC, New York, NY, USA) module of the Schrodinger suite 2014-1 (Schrödinger, LLC, New York, NY, USA). The rotamers of the conserved amino acid residues are preserved in the homology model in a way that the final 3D model does not significantly deviate from the template structure. The stereochemical quality of the model was verified using MolProbity [33]. Further detail on the methodology based on Molecular Modeling has been described in a previous study [51].

### 4.5. Docking

The chemical structures of the neonicotinoids IMI and THI are shown in Figure 1. The structures were converted to 3D at pH 7.0 ± 0.2 using the LigPrep v3.0 (Schrödinger, LLC, New York, NY, USA) module of the Schrodinger suite 2014-1 (Schrödinger, LLC, New York, NY, USA). The confgen program (Schrödinger, LLC, New York, NY, USA) was then used to retrieve the lowest energy conformer of each insecticide for docking. The docking was carried out using the Glide v6.3 [52] program of the Schrodinger suite 2014-1 (Schrödinger, LLC, New York, NY, USA). The active site was defined by all residues located in a sphere of 6 Å centered on the ligand. The same residues were selected for receptor grid generation. The extra-precision (XP) [53] mode of the docking algorithm was employed to dock IMI and THI ligands. It is worth remembering that nAChRs are organized as pentamers, that is, there are five identical ligand binding sites and the ligand binds between the cleft of the two subunits. The ligands were therefore docked in all the five interfaces of the cockroach Pameα7 nAChR model. The docking results were validated by comparing the predicted ligand binding modes to the crystallographic Ac-AChBP-neonicotinoid structures.

### 4.6. Statistical Analysis

Data were shown as mean ± SEM and analyzed using Prism 7 (GraphPad Software, La Jolla, CA, USA). Responses to experimental neonicotinoid applications were determined relative to the preceding nicotine control responses in the same experimental conditions. The dose response curves were derived from the fitted curve following the equation: Y = Imin + (Imax − Imin)/(1 + 10(log(EC50 X)H)), where Y is the normalized response, Imax and Imin are the maximum and minimum responses, H is the Hill coefficient, EC_50_ is the concentration giving half the maximum response and X is the logarithm of the compound concentration.

## Data Availability

All data from the present study can be found from the corresponding authors.

## Supplementary Materials

The following are available online at https://www.mdpi.com/article/10.3390/ijms22189880/s1.
