# Supplementary material for: Mode of Action of Neonicotinoid Insecticides Imidacloprid and Thiacloprid to the Cockroach Pameα7 Nicotinic Acetylcholine Receptor
Source: Int J Mol Sci. 2021 Sep 13;22(18):9880. doi: 10.3390/ijms22189880 (PMC8471617; doi:10.3390/ijms22189880)
Supplement: Supplementary file 1 [file ijms-22-09880-s001.zip › ijms-1349353-supplementary.pdf]

Supplementary Materials

Mode of action of neonicotinoid insecticides imidacloprid and thiacloprid to the cockroach Pameα7 nicotinic acetylcholine receptor

Alison Cartereau, Emiliane Taillebois, Jean-Yves Le Questel and Steeve H. Thany

Table S1. Selected interatomic distances, docking scores (DS) and glide energies (GE) (kcal mol^{-1}) of IMI and THI binding in cockroach Pameα7 nAChR (CD subunits interface).

Neonicotinoid	Interaction	Interaction distance (Å)	DS	GE
	N5…H(O) (W248)	2.01		
	W248 O…HN(Val159 C)^{MC}	2.13		
	W248(O)H…OC(Asn147 C)^{MC}	2.11		
	Ctr$_1^a$…Ctr(5) (Trp 189 D)^{SC}	5.24		
	C9H… Ctr(5) (Trp 189 D)^{SC}	3.01		
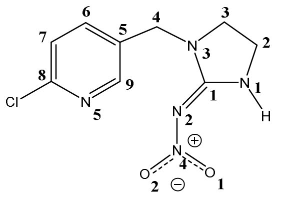	C4H…S (Cys 233 D)^{SC}	2.64	-7.2	-75.1
	Ctr$_2^b$…Ctr(6) (Trp 189 D)^{SC}	4.10		
IMI	C3H… Ctr(6) (Trp 189 D)^{SC}	2.34		
	Ctr$_2^b$…Ctr(6) (Tyr 231 D)^{SC}	3.80		
	C3H… Ctr(6) (Tyr 231 D)^{SC}	2.79		
	O2…H(N) (Lys 97 D)^{SC}	4.29		
	N4…H(O) (W248)	1.99		
	W248 O…HN(Val159 C)^{MC}	2.13		
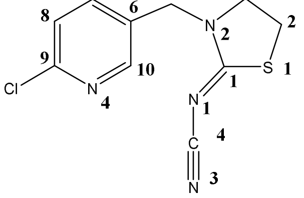	W248(O)H…OC(Asn147 C)^{MC}	2.11		
	Ctr$_1^a$…Ctr(5) (Trp 189 D)^{SC}	5.18		
THI	C10H… Ctr(5) (Trp 189 D)^{SC}	3.00		
	C5H…S (Cys 233 D)^{SC}	2.67	-4.6	-71.7

Ctr_2^b…Ctr(6) (Trp 189 D) ^{SC}	4.12
C2H… Ctr(6) (Trp 189 D) ^{SC}	2.93
C3H… Ctr(6) (Trp 189 D) ^{SC}	2.88
Ctr_2^b…Ctr(6) (Tyr 231 D) ^{SC}	3.89
C3H… Ctr(6) (Tyr 231 D) ^{SC}	2.36

[a]Ctr1 : centroid of the pyridinic ring of IMI and THI; Ctr2: centroid of the five membered saturated ring of IMI and THI; Ctr(6) correspond to the centroid of the six or six membered ring of the indole lateral chain of Trp or of the phenole ring of Tyr whereas Ctr refers to the centroid of the five membered of Trp; ^{MC} main chain of the residue; ^{SC} sidechain of the residue.
